# REFRAMING AGING: THROUGH THE LENS OF ETHICAL ARTIFICIAL INTELLIGENCE

**DOI:** 10.1093/geroni/igae098.3888

**Published:** 2024-12-31

**Authors:** Cynthia McNellis

**Affiliations:** American Public University System, Egg Harbor Twp, New Jersey, United States

## Abstract

Artificial Intelligence (AI) technology is advancing at a rapid pace. In addition, our older adult cohort is growing at an unprecedented rate. The purpose of my research is to address the ethical concerns when AI intersects with this population, offering policy recommendations to bridge this ethical digital divide. To address this AI ethical dilemma, my research did not reach back prior to 2019 in order to provide a concise policy analysis that includes existing AI ethical policies/recommendations from the European Union, United States, and U.S. States, key differences, and how ethical AI policies are central to Area Agencies on Aging’s development of aging services. My findings indicated gaps in ethical AI design with developed policy recommendations. These gaps include inclusive data collection, user-centric design, algorithmic biases and understanding, digital literacy, New Jersey AI Task Force membership, and a need for a New Jersey AI Ethical Framework. With my five policy recommendations, New Jersey will be better positioned in assuring AI systems will be equitable, ethical, and effective in addressing the present and future needs of older adults. With this ethical AI framework, New Jersey will help to better older adults’ quality of life, as well as save valuable time and resources through the new industrial revolution that is Artificial Intelligence. Keywords: Artificial Intelligence, ethics, older adults, algorithmic bias, AI ageism

